# Functional diversification of the MADS-box gene family in fine-tuning the dimorphic transition of *Talaromyces marneffei*

**DOI:** 10.1128/msystems.00464-25

**Published:** 2025-06-03

**Authors:** Xueyan Hu, Yun Zhang, Juan Wang, Minghao Du, Yang Yang, James J. Cai, Ence Yang

**Affiliations:** 1 Department of Microbiology & Infectious Disease Center, School of Basic Medical Sciences, Peking University Health Science Center 33133, Beijing, China; 2 Department of Medical Bioinformatics, School of Basic Medical Sciences, Peking University Health Science Center 33133, Beijing, China; 3 Department of Biochemistry and Molecular Biology, Peking University Health Science Center 33133, Beijing, China; 4 Department of Veterinary Integrative Biosciences, Texas A&M University 14736, College Station, Texas, USA; University of Massachusetts Amherst, Amherst, Massachusetts, USA

**Keywords:** *Talaromyces marneffei*, MADS-box, dimorphism transition, RNA-seq, ChIP-seq, fungal adaptation

## Abstract

The dynamic transition between yeast and hypha is a crucial adaptive mechanism for many human pathogenic fungi, including *Talaromyces marneffei*, a thermodimorphic fungus responsible for causing fatal talaromycosis. In the current study, we elucidated the roles of the MADS-box family in fine-tuning the dimorphic transition in *T. marneffei* through functional diversification of members. Utilizing adaptive laboratory evolution, we identified an enrichment of MADS-box genes in mutants deficient in yeast-to-mycelium transition. Further phylogenetic analyses revealed a significant expansion of the MADS-box gene family within *T. marneffei*. Functional genetic manipulations revealed that overexpression of *mads9*, as opposed to its paralog *mads10*, effectively delayed the hypha-to-yeast transition. Through integrating RNA sequencing and chromatin immunoprecipitation sequencing, we demonstrated that *mads9* and the previously characterized *madsA* (*mads7*) modulated the rate of hypha-to-yeast conversion by orchestrating metabolic pathways and membrane dynamics, respectively, with mutual regulation via shared target genes. Our findings illuminated the distinct functional roles of the MADS-box family in regulating dimorphic transitions in *T. marneffei*, offering new insights into fungal adaptability.

**IMPORTANCE:**

The dimorphic transition between yeast and hyphal forms in *Talaromyces marneffei* is a critical adaptive mechanism that underpins its pathogenicity, particularly in response to environmental cues such as temperature. In this study, we elucidated the role of the MADS-box transcription factor family and discovered that its members collaboratively regulate dimorphic transitions by assuming distinct roles in the morphogenesis, enhancing the understanding of the thermal adaptation of *T. marneffei* and the functional roles of the MADS-box gene family outside the plant.

## INTRODUCTION

Human pathogenic fungi, predominantly environmental in origin, often undergo morphological transitions between yeast/yeast-like and hypha/hyphal-like forms to adapt to the human body temperature during infection processes (1, 2). Understanding the genetic mechanisms behind these transitions across environments and hosts can provide valuable insights into managing current fungal pathogens and preventing the emergence of new ones (3, 4). *Talaromyces marneffei* (previously known as *Penicillium marneffei*) is an opportunistic human pathogen that poses a significant health risk in immunocompromised individuals, particularly in Southeast Asia and South China (5). Unlike the other species in its genus, *T. marneffei* is capable of thermal dimorphism, switching between saprophytic hyphae (25°C) and pathogenic yeast forms (37°C) in response to temperature changes (6). The morphogenetic transition from hyphae to yeast in *T. marneffei* exhibited temperature-dependent kinetics, initiating at 32°C and reaching maximal yeast production at 37°C, consistent with its characteristic thermodimorphic behavior (7). Since these morphological shifts can be fully induced by temperature in the laboratory (8), *T. marneffei* serves as an ideal model for studying the mechanisms of heat adaptation in human pathogenic fungi.

Previous studies have demonstrated that a crucial aspect of thermal adaptation in *T. marneffei* is its temperature-regulated dimorphic transition between hyphal and yeast forms (1). This complex phenotypic change involves alterations in multiple genes and pathways, highlighting the intricate regulatory mechanisms at play (6). Among the key regulators identified are the *abaA* (9), *areA* (10), *hgrA* (11), *madsA* (*mads7*) (12), and *msgA (*13) genes. During the hypha-to-yeast transition, mutations in the ATTS transcription factor (TF) gene *abaA* can lead to growth defects when conidia produced by hyphae convert to yeast. Conversely, during the yeast-to-hypha transition, overexpression of the C2H2 transcription factor gene *hgrA* inhibits yeast growth at 37°C, while overexpression of the MADS-box transcription factor gene *madsA* promotes this conversion at 37°C. During yeast morphogenesis *in vivo*, the GATA factor gene *areA* and the Dbl homology/BAR domain gene *msgA* facilitate or maintain yeast cell growth within the host. Notably, among these genes, *madsA* is a member of the MADS-box family, which has been extensively studied for its regulatory roles in plants. However, the role of the MADS-box family in fungal morphogenesis remains underexplored.

The MADS-box gene family consists of transcription factors that are prevalent in eukaryotic organisms (14). This family is named after its initial members: MCM1 from *Saccharomyces cerevisiae* (15), AGAMOUS from *Arabidopsis thaliana* (16), DEFICIENS from *Antirrhinum majus* (17), and SRF from *Homo sapiens* (18). All MADS-box members share a conserved domain that is crucial for nuclear localization, DNA binding, protein dimerization, and interactions with accessory factors (19–21). MADS-box transcription factors participate in a spectrum of biological processes. In plants and animals, these factors regulate key cell proliferation and differentiation and are significant for muscle morphogenesis (22–25). Notably, a large number of MADS-box transcription factors in flowering plants interact with each other and constitute a complex regulatory network that determines floral organ morphology (26). In fungi, such as *Schizosaccharomyces pombe*, *Aspergillus fumigatus,* and *Candida albicans*, several MADS-box genes are involved in cellular morphogenesis, responses to environmental signals, and maintenance of cell wall integrity (27–30). Our previous studies on *T. marneffei* identified and validated the function of the *madsA* gene, where loss of *madsA* resulted in a faster transition from yeast to mycelium (12, 31). Additionally, we observed significant gene duplication within the MADS-box transcription factor family, highlighting the need for further investigation into how this family contributes to the dimorphic transition.

In this study, through temperature-driven adaptive laboratory evolution of *T. marneffei* strains exhibiting hyphal formation defects, we observed strong selection pressure favoring activation of MADS-box transcription factors. We then generated overexpression and gene knockout mutants of these MADS-box genes using genetic manipulation, confirming their distinct regulatory roles in the biphasic conversion of *T. marneffei*. Through RNA sequencing (RNA-seq) and chromatin immunoprecipitation sequencing (ChIP-seq), we found that the MADS-box transcription factors *mads9* and *madsA* (annotated as *mads7* in this phylogenetic family identification) regulate different downstream processes via distinct TF binding sites, while also sharing common target genes in fine-tuning dimorphic transition. The functional divergence of *mads9* and *madsA* emphasizes the importance of the MADS-box family in understanding morphological transformation and adaptive evolution of *T. marneffei*.

## RESULTS

### The MADS-box gene family is enriched in dimorphic transition-deficient mutants derived from adaptive laboratory evolution in *Talaromyces marneffei*

**Fig 1:**
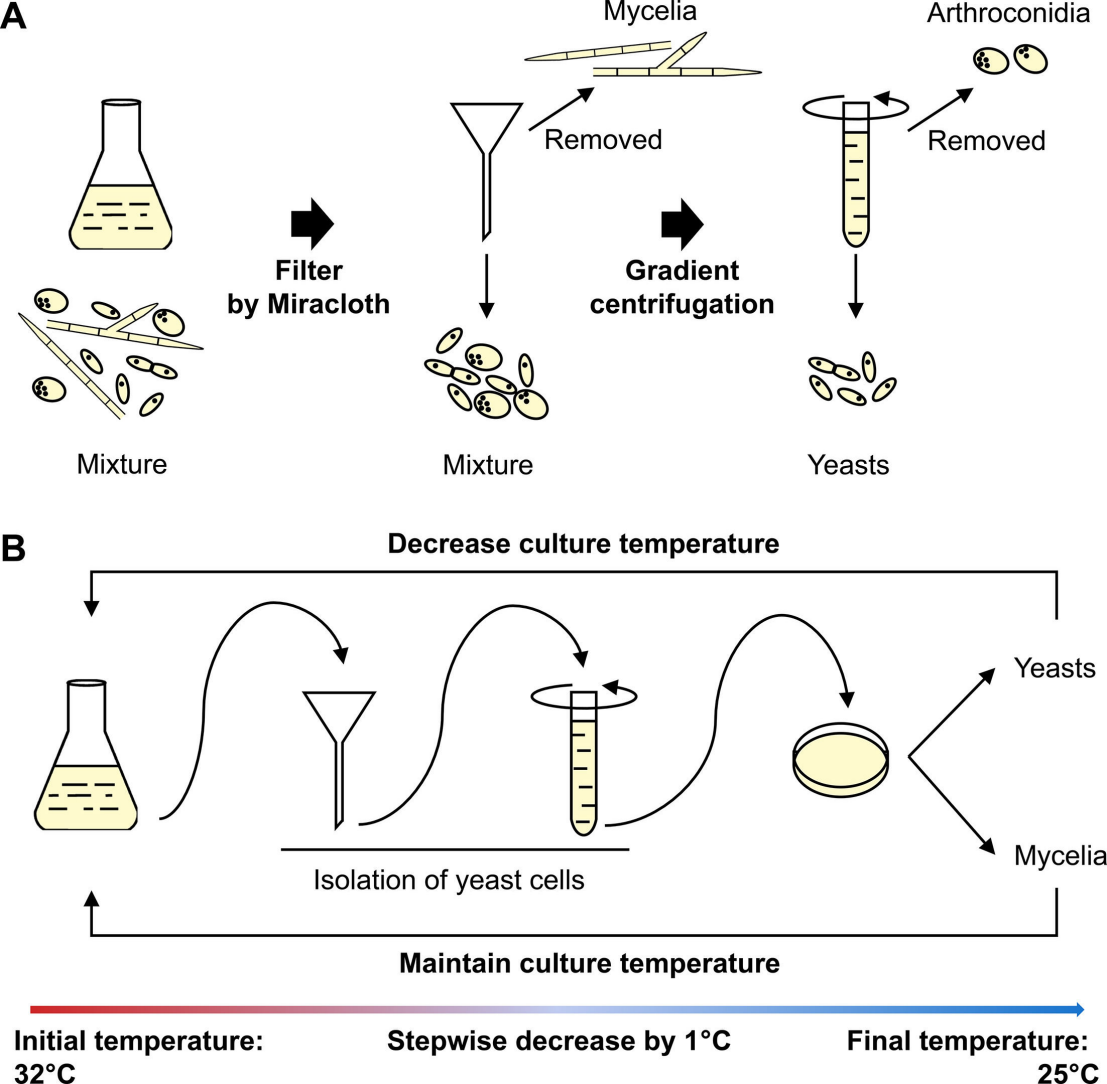
Adaptive laboratory evolution inducing dimorphic transition defective strains of *T. marneffei* PM1. (**A**) Schematic workflow for isolating three different morphological cell types: mycelium, yeast, and conidia. Mycelium was filtered out using four layers of Miracloth, followed by centrifugation to separate yeast cells from conidia. (**B**) Flowchart of the experimental evolution inducing dimorphic transition defective strains of *T. marneffei*. Cells from the Sabouraud dextrose broth (SDB) liquid medium were collected, and yeast cells were isolated using the method depicted in (A) before being inoculated onto Sabouraud dextrose agar (SDA) plates to confirm their growth morphology. If substantial mycelium was present, cultures were maintained at the original temperature in SDB; if the predominant form was yeast, the incubation temperature was lowered by 1°C, continuing in SDB. This selection process was repeated until the culture temperature was reduced to 25°C.

To better understand the regulatory mechanisms underlying dimorphic transition, we first generated a population of transition-deficient mutants by employing adaptive laboratory evolution with morphotype separation (Fig. 1A and B). Utilizing bulked segregant analysis combined with high-throughput sequencing, we identified 426 single-nucleotide variants and 200 indels from the transition-deficient population (Fig. S1A). The distribution of mutation frequency indicated that the transition-deficient population was composed of multiple mutants rather than a single mutated strain (Fig. S1B). Among the 31 completely mutated sites, there were only six mutants that resulted in alterations to the protein coding sequences, including three frameshift and three nonsynonymous variants, with half being related to transmembrane transport (Table S1).

We identified 792 structural variations, with five exhibiting mutation frequencies exceeding 80% (Fig. S1C). Given that the dimorphism-defective phenotype suggests a loss of function, we focused on the impact of three deletions among these high-frequency variants and identified 54 deleted genes. Functional enrichment analysis of the deleted genes revealed significant associations with Gene Ontology (GO) functions such as protein dimerization activity (*P* = 4.0 × 10^−4^), protein kinase activity (*P* = 1.8 × 10^−3^), and DNA binding (*P* = 3.1 × 10^−3^). Interestingly, the MADS-box transcription factor superfamily (*P* = 1.6 × 10^−5^) was significantly enriched in the IPR domain, with MADS genes (*mads9*, *mads10*, and *mads13*) identified within the deletion fragments. Combined with our previous findings (12, 31), we hypothesize that multiple members of the MADS-box family may be involved in the dimorphic transition of *T. marneffei*.

### Phylogenetic analysis of the MADS-box gene family in *Talaromyces* genus

**Fig 2:**
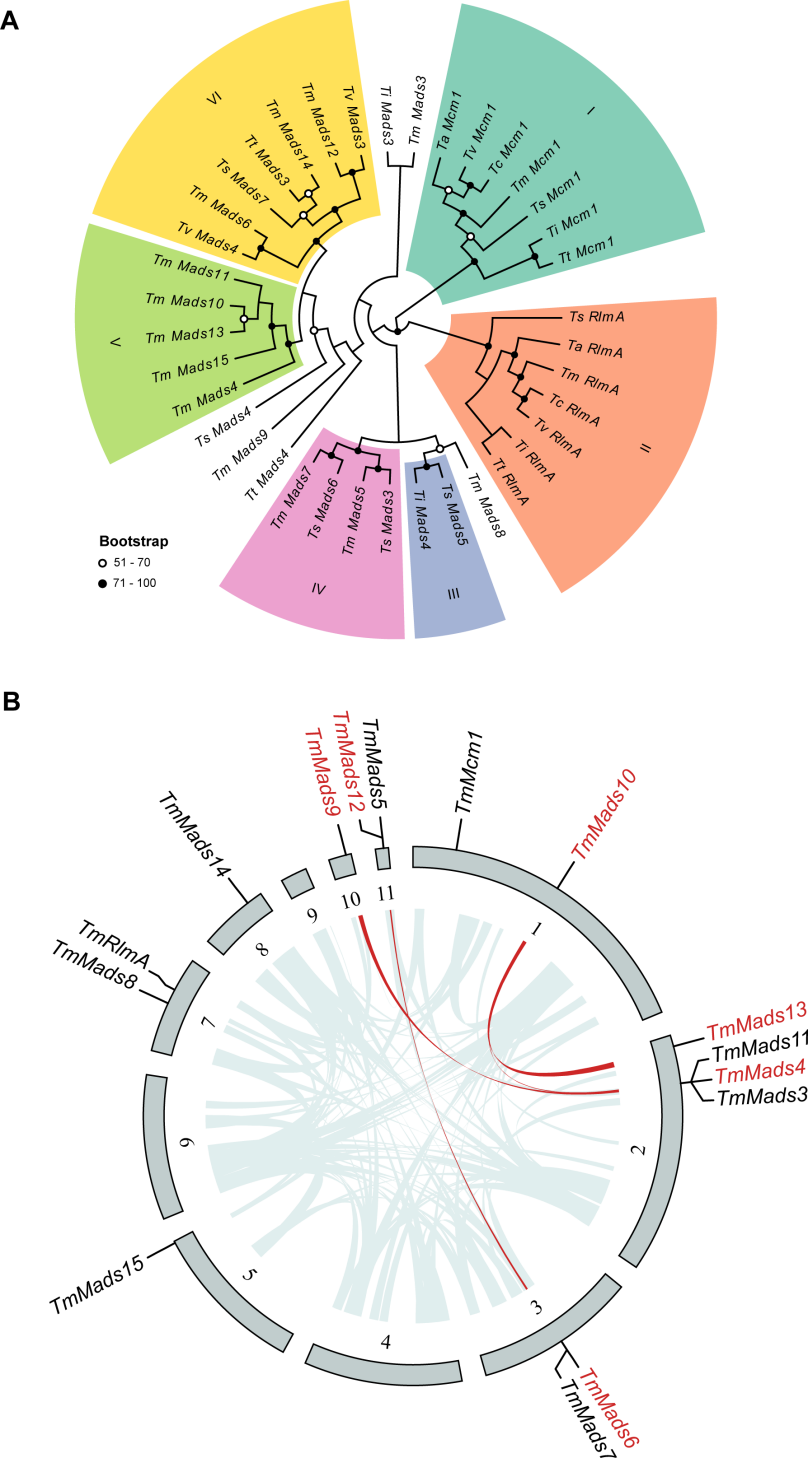
Phylogenetic analysis and synteny of the MADS-box family in *T. marneffei*. (**A**) Phylogenetic tree of the MADS-box gene family in the *Talaromyces* genus. The type of point (solid or hollow) represents the support rate. (**B**) Synteny analysis of the MADS-box gene family in *T. marneffei*. The outer layer represents the genomic sequences of wild-type strain PM1, with the locations of all MADS-box transcription factors annotated. The inner layer indicates the syntenic regions of the PM1 genome, with red highlighting the syntenic regions containing MADS-box transcription factors.

To explore the potential regulatory role of the three identified MADS-box genes above (*mads9*, *mads10*, and *mads13*) in dimorphic transition, we performed a comprehensive phylogenetic analysis of the MADS-box gene family across seven *Talaromyces* species (Table S2). Through the IPR protein domain database, we identified a total of 38 genes containing at least one MADS-box domain across genomes of these species. The *T. marneffei* PM1 strain exhibited the highest number with 15 MADS-box family genes, followed by *Talaromyces stipitatus* with 7. *Talaromyces islandicus*, *Talaromyces verruculosus*, and *Talaromyces atroroseus* each possessed four genes, while *Talaromyces cellulolyticus* and *Talaromyces amestolkiae* each contained two genes (Fig. 2A; Table S2).

Phylogenetic analysis grouped 33 MADS-box genes into six main distinct clades (Fig. 2A). Clades I and II corresponded to the highly conserved eukaryotic MADS-box transcription factors *Mcm1* (15) and *RlmA* (29). In the *T. marneffei* PM1 strain, five genes—*mads4*, *mads10*, *mads11*, *mads13*, and *mads15*—formed a monophyletic clade, suggesting a latest common ancestry. Further analysis of the *T. marneffei* PM1 strain revealed several collinear regions, including those between the *mads4* and *mads10* (eight gene pairs), *mads10* and *mads13* (six gene pairs), *mads4* and *mads9* (seven gene pairs), and *mads6* and *mads12* (six gene pairs) (Fig. 2B; Fig. S2).

Based on these collinear regions, the mechanisms responsible for the expansion of the MADS-box gene family in *T. marneffei* were inferred by MCScanX-transposed (32). The species-specific increase in MADS-box transcription factors appeared to occur through two primary mechanisms: segmental duplication and transposition-mediated duplication. For instance, m*ads4*, *mads9*, *mads10*, and *mads13* may have originated from the segmental duplication of a common ancestral gene, while *mads6* and *mads12* likely arose from the segmental duplication of another ancestral gene. In contrast, the three genes, *mads11*, *mads14*, and *mads15*, seem to have resulted from independent transposition-mediated duplication events. Although *mads9* did not form a monophyletic branch with *mads10* and *mads13*, it is hypothesized that these three genes may have originated from the segmental duplication of a shared ancestral sequence (Fig. 2B). This suggests that segmental duplication has played a pivotal role in the diversification and expansion of the MADS-box gene family, contributing to the adaptive evolution of dimorphic transition in *T. marneffei*.

### MADS-box family involved in regulating morphogenesis of *T. marneffei* with functional differentiation

**Fig 3:**
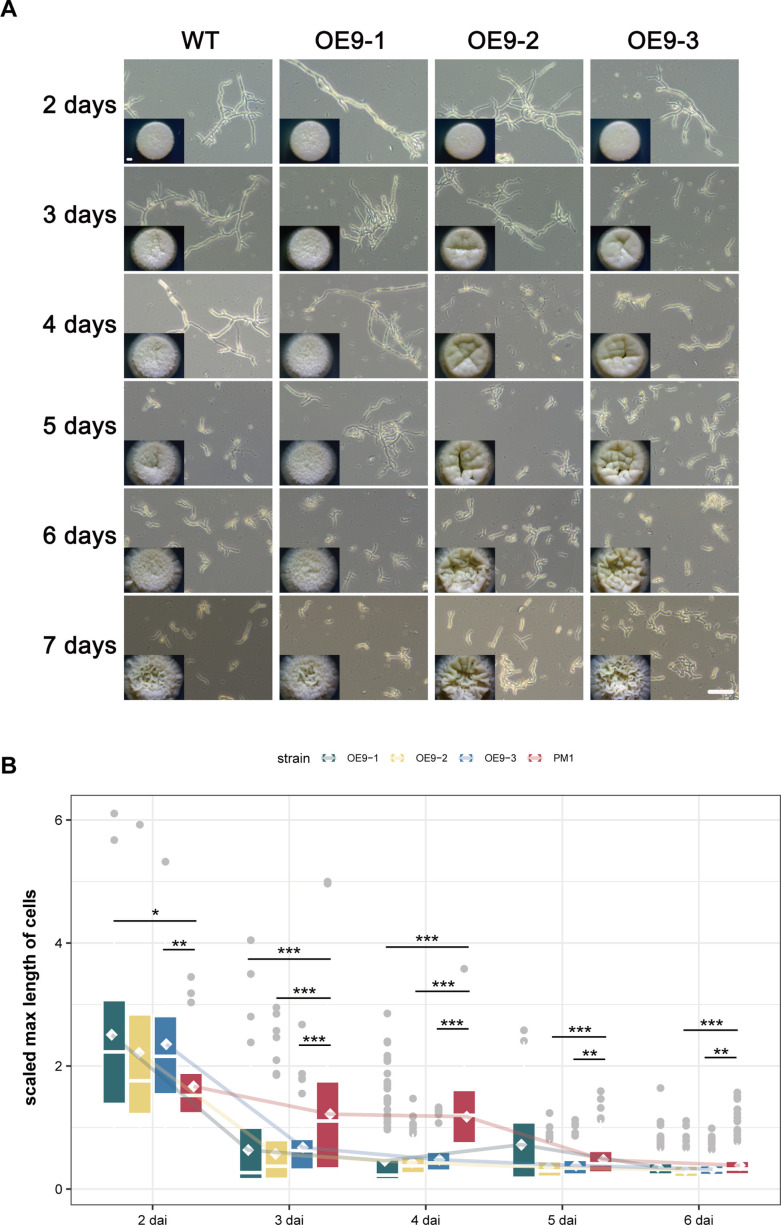
Overexpression of *mads9* promoted morphogenesis of yeast cells in *T. marneffei*. (**A**) Microscopic images were acquired under 20× objective lens with phase contrast effect. Bar = 20 µm. Time-course phenotypes of colonies grown on SDA plates under 37°C were shown as insets. Bar = 1 mm. (**B**) Comparison of cell length during static culture at 37°C. Comparisons among different groups were performed using Wilcoxon rank-sum tests. Box plots represent median values and upper quartile (Q3) and lower quartile (Q1). Diamond dots represent mean values. ***, *P* < 0.001; **, *P* < 0.01; *, *P* < 0.05.

Based on the potential monophyletic origin of *mads9*, *mads10*, and *mads13*, we further investigated the roles of *mads9* and *mads10* in regulating the morphogenesis of *T. marneffei*, considering the low expression level of *mads13* in our previous data (33). We first constructed overexpression mutants for *mads9* and *mads10* and analyzed their growth at 37°C and 25°C conditions, respectively (Fig. S3A). At 2 days after incubation (dai) at 37°C, OE-*mads9* had significantly longer arthrospores relative to wild-type PM1 strain arthrospore morphology (Fig. 3A and B). However, at 3 dai, there was a significant decrease in length, shifting toward a short rod-shaped yeast morphology, which suggested a significant acceleration of yeast production (Fig. 3A and B). By 4 dai, colonies of the OE-*mads9* strain appeared fluffier and smoother, whereas those of the wild-type were generally flat with more spikes. Most of the wild-type PM1 cells exhibited elongated and branched hyphal-like growth, whereas abundant shorter and yeast-like cells were observed in the OE-*mads9* strains (Fig. 3A and B). As the time increases, the difference between OE-*mads9* and wild-type decreased (Fig. 3A and B). By 7 dai, the OE-*mads9* strains exhibited more wrinkled colony surfaces than the wild-type, which is generally indicative of excessive cell proliferation (Fig. 3A). The above results suggest that high expression of *mads9* may induce abnormalities in yeast cell morphogenesis. We also performed the same examination of OE*-mads10*. In contrast, OE-*mads10* strains showed no significant morphological changes in comparison with the wild-type strains. We also performed growth analysis at 25°C, and all strains grew in a filamentous form with normal spore formation as indicated by the yellow-green colonies, with no obvious differences between the wild-type and OE-*mads9* strains (Fig. S4A), indicating that the high expression level of *mads9* specifically interfered with the morphogenesis of yeast cells without affecting hyphal development.

Then, we generated knockout strains of *mads9* and *mads10* to explore the effects of loss of function of these genes (Fig. S3B and C). We monitored the growth of each genotype from 2 to 6 days after inoculation. No significant phenotypic changes were observed in the KO*-mads9,* KO*-mads10,* and KO*-mads9/mads10* knockout lines at either 37°C or 25°C compared to the wild-type PM1 strain (Fig. S4B and C). This phenomenon may be due to the functional redundancy among MADS-box family genes (34).

Together, these findings suggest that *mads9* may participate in promoting yeast cell formation during constant growth at 37°C, while its homologous gene *mads10* did not have such roles.

### *mads9* regulates the dynamic dimorphic transition of *T. marneffei*

Since static culture conditions are unable to accurately capture the dynamic transformation of hypha-to-yeast morphology during the dimorphic transition, we conducted a mycelium-to-yeast (M-to-Y) transition experiment to further evaluate the role of MADS-box genes in this process. At the colony level, no significant differences were observed between the wild-type PM1 strains and the *mads9* knockout and overexpression strains (Fig. S5A). However, due to the inherent heterogeneity of the colonies—such as the presence of cells in different developmental states at the center versus the edge—sampling variability introduced unavoidable interference. To overcome this limitation, we then developed a dynamic microscopic analysis of the liquid mycelial cultures, a more homogeneous culture that allowed us to focus on changes at the cellular level.

**Fig 4:**
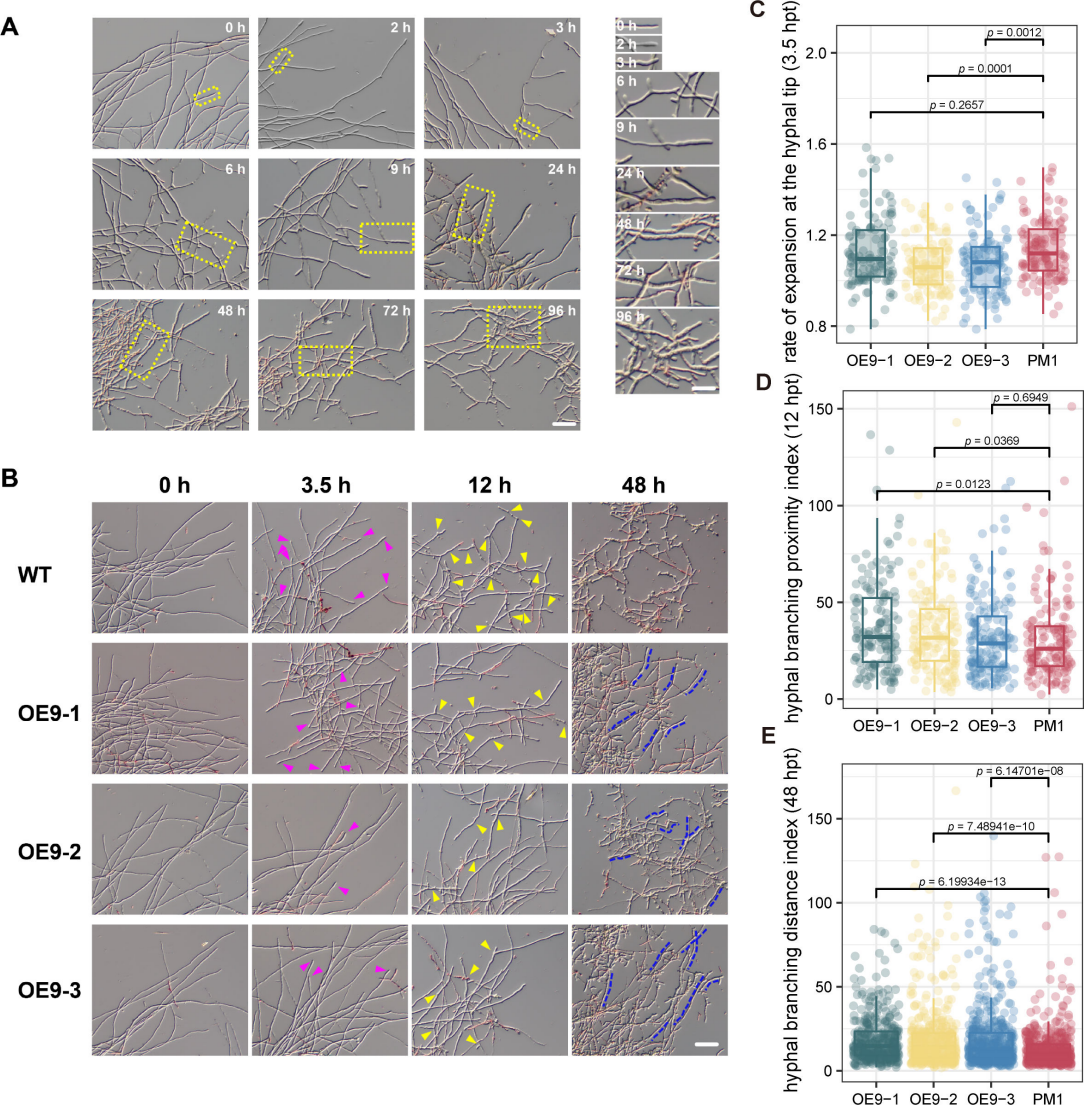
Overexpression of *mads9* delayed the M-to-Y transition in *T. marneffei*. (**A**) Time-course microscopic analysis of the morphological changes in the wild-type cells grown in SDB transferred from 25°C to 37°C. Bar in the left panel is 50 µm. The highlighted region of interest in the left panel was magnified artificially and shown in the right panel. Bar = 25 µm. (**B**) Microscopic analysis of the morphological changes in the wild-type and the OE-*mads9* cells grown in SDB transferred from 25°C to 37°C at indicated time points. Purple arrowheads indicated the swelling cell tips. Note that the ratio of swelling tips to the total cell ends visible in the wild-type was bigger than those in the OE-*mads9* strains. Yellow arrowheads pointed toward the bumpy cell surfaces with newly formed protrusions. Blue dotted lines labeled the longer cell remained in the OE-*mads9* strains. Bar = 50 µm. (**C**) The rate of expansion at the hyphal tip at 3.5 hpt of OE-*mads9* and wild-type PM1. The rate of expansion at the hyphal tip describes the speed at which the leading edge of a fungal hypha grows and extends, reflecting the dynamic processes of cell wall synthesis and extension during fungal growth. The upper and lower boundaries of the box represent the upper quartile (Q3) and lower quartile (Q1) of the data. The line in the middle of the box represents the median. Whiskers represent the minimum and maximum non-outlier values extending from the box to the data (interquartile range = Q3 − Q1). (**D**) Hyphal branching proximity index at 12 hpt of OE-*mads9* and wild-type PM1. The hyphal branching proximity index measures the closeness of branching points along the fungal hyphae, shedding light on the spatial arrangement and growth patterns. (**E**) Hyphal branching distance index at 48 hpt of OE-*mads9* and wild-type PM1. The hyphal branching distance index quantifies the average distance between branching points on fungal hyphae, offering insights into growth dynamics and network expansiveness.

Initially, we examined the time-course morphological transformation of the wild-type PM1 strain under a microscope to establish a baseline for normal morphological transitions (Fig. 4A). Before the M-to-Y transition began, most elongated mycelia exhibited typical even cell tips. At 2 h post-transfer (hpt), many cell tips swelled like partially inflated balloons. By 3 hpt, these swollen regions expanded, with obvious branching at the mycelial ends and new tips forming around 6 hpt. Over time, these swollen regions continued to expand, becoming distinct from older, thinner mycelia. From 9 hpt onward, swollen mycelial ends became predominant in the culture, with older reddish dying cells or debris easily distinguished. At 24 hpt and beyond, differential interference contrast microscopy revealed bumpy and irregular cell surfaces. Active vesicles and septation were visible within cells, with contents appearing condensed.

To analyze the morphological changes over time, characterized by the expansion, branching, and eventual death or fragmentation, we conducted a similar time-course morphological analysis on the mutant strain. To quantitatively assess this process, we introduced three metrics at specific time points: the rate of expansion at the hyphal tip, the hyphal branching proximity index, and the hyphal branching distance index. Upon transition from 25°C to 37°C, OE-*mads9* strains showed a clear delay in the M-to-Y transition under the microscope (Fig. 4B), though not in the colonies (Fig. S5A). At 3.5 hpt, fewer cells exhibited swelling tips in OE-*mads9* strains compared to wild-type (Fig. 4B and C). By around 12 hpt, most wild-type cell ends displayed active branching with numerous new tips forming; however, OE-*mads9* strain cell tips only began significant swelling at this point with fewer branches (Fig. 4B and D). As the transition progressed to around 48 hpt, condensed cells with bumpy surfaces interspersed with dying cells were observed in wild-type strains. In contrast, OE-*mads9* cells remained longer and less condensed (Fig. 4B and E), indicating a transition delay compared to wild-type PM1 cells. Consistent with the findings from static culture, no significant differences were observed between the KO-*mads9* mutant and the wild-type strain in the time-course experiment (Fig. S5).

Overall, these results indicate that *mads9* was involved in regulating the dimorphism of *T. marneffei*, where high expression induces aberrant morphogenesis and delayed mycelia-to-yeast transition.

### Certain MADS-box family genes fine-tune dimorphic transitions through functional differentiation

**Fig 5:**
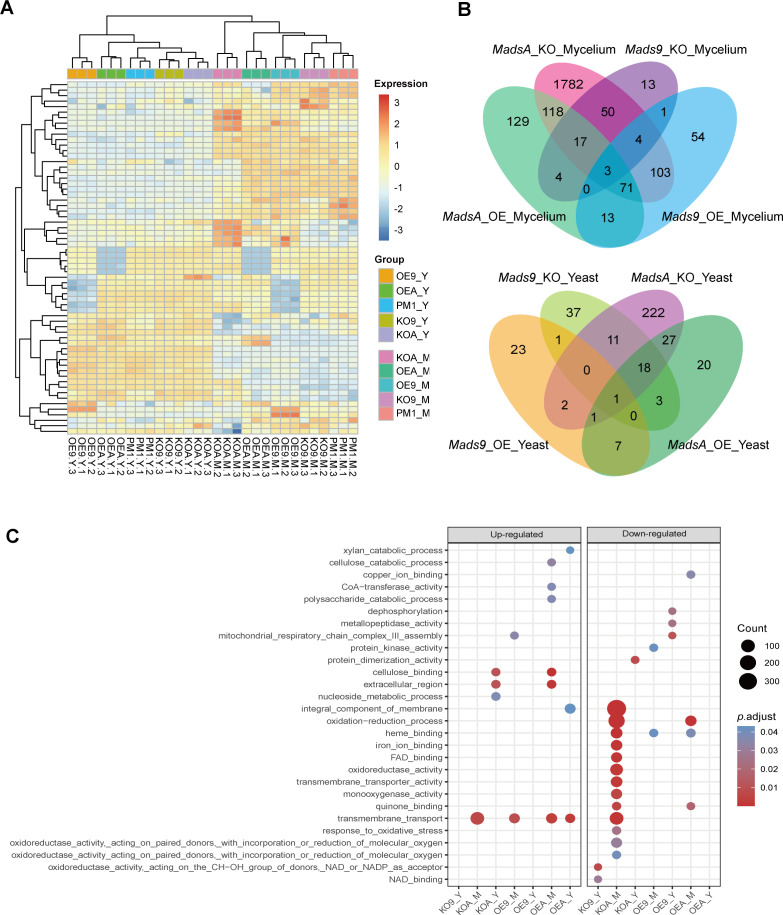
Differential gene expression analysis of MADS-box mutant strains in *T. marneffei.* (**A**) RNA-seq gene expression clustering of mutant and wild-type strains. The color code of the heatmap represents the *z*-scored gene expression level. The correlation of gene expression patterns and levels between biologically repeated samples was consistent. (**B**) Differential gene expression analysis of *madsA* and *mads9* mutant strains. The Venn diagram displayed the count of unique and common differentially expressed genes (DEGs) between mutant strains and PM1 under two conditions. (**C**) Enrichment results of differentially expressed genes in the mycelial and yeast phase. The size of the point represents the count of DEGs, and the gradual color change from red to blue represents the adjusted *P*-value change from low to high.

Given that both *mads9* and our previously studied *madsA (*12) (annotated as *mads7* in this phylogenetic family identification) regulate dimorphic transitions with opposing trends, i.e., *mads9* delays yeast development in dimorphic transition, while *madsA* promotes hyphal development in dimorphic transition, we next investigated the regulatory mechanisms of the MADS-box gene family to enhance our understanding of their roles in temperature adaptation of *T. marneffei*. We performed RNA sequencing on the *mads9* and *madsA* mutants, which exhibited the most significant phenotypes, as well as on the wild-type PM1 strain under both mycelial and yeast growth conditions. Hierarchical clustering revealed consistent gene expression patterns across biological replicates, indicating high reproducibility of the sequencing data (Fig. 5A).

Under hyphal conditions, differential expression analysis using DESeq2 identified 249, 92, 355, and 2,148 differentially expressed genes (DEGs) in the OE-*mads9*, KO-*mads9*, OE-*madsA*, and KO-*madsA* strains, respectively (Fig. 5B). The *mads9* mutant displayed an expression profile more similar to the wild-type than to the *madsA* mutant under both mycelial and yeast conditions. This may indicate that the mode of action of *mads9* did not involve a broad transcriptional regulatory effect, which may account for the lack of statistical significance observed in the phenotypic assays. GO enrichment analysis provided insights into the biological processes associated with the differentially expressed genes. In the *mads9* mutants, enriched GO terms included “transmembrane transport,” “protein kinase activity,” and “mitochondrial respiratory chain complex III assembly.” Meanwhile, the *madsA* mutants were enriched for terms related to “membrane integral components,” “oxidation-reduction processes,” and “iron ion binding” (Fig. 5C). Under yeast conditions, 35, 71, 77, and 282 DEGs were identified in the OE-*mads9*, KO-*mads9*, OE-*madsA*, and KO-*madsA* strains, respectively (Fig. 5B). In this condition, the *mads9* mutants were enriched for terms such as “mitochondrial respiratory chain complex III assembly,” “dephosphorylation,” “NAD binding,” and “metallopeptidase activity.” On the other hand, the *madsA* mutants showed enrichment in terms including “transmembrane transport,” “protein dimerization activity,” and “cellulose binding” (Fig. 5C).

These results indicated that the overexpression of the *mads9* gene may impact energy metabolism and cellular signaling pathways, thereby inhibiting the transition from hyphal to yeast form. On the other hand, *madsA* overexpression promotes hyphal development by modulating membrane integrity, redox balance, and ion homeostasis. The expansion of the MADS-box gene family may provide a genetic basis for the fine-tuning of dimorphic transitions in *T. marneffei*, as exemplified by the roles of *mads9* and *madsA* in adapting the fungus to different growth forms.

### Regulation of common dimorphic transition effector genes between MADS-box families

**Fig 6:**
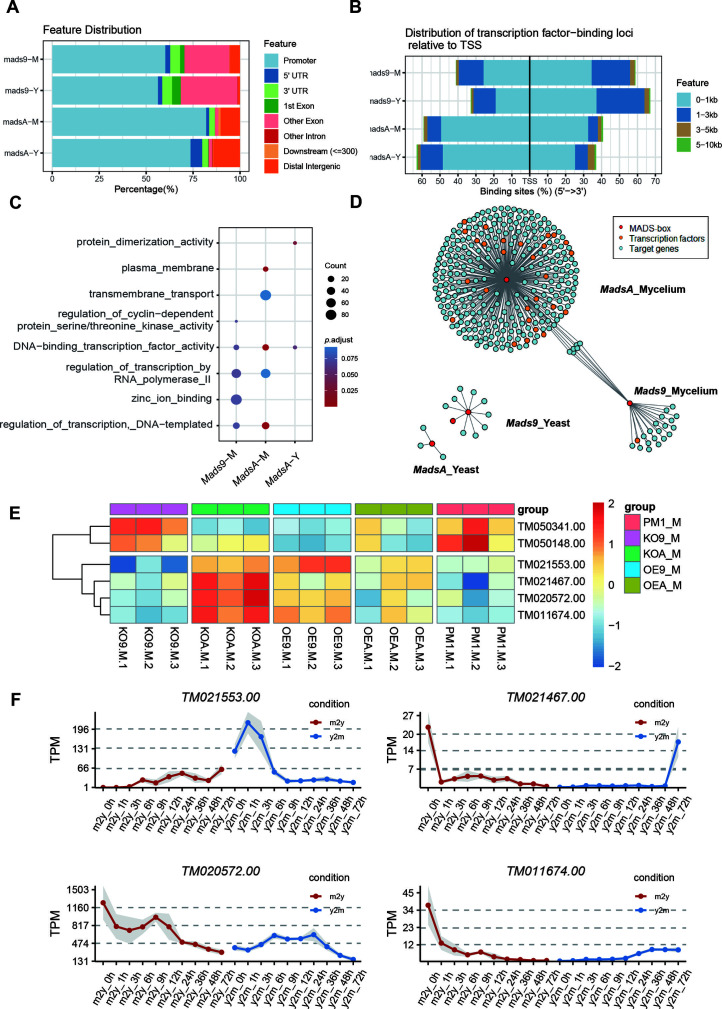
Gene regulatory network of the MADS-box family. (**A**) Genome-wide binding patterns of MADS-box transcription factors. The ChIP-seq peaks are divided into several categories: promoter, first exon, other exons, first intron, other introns, 5´ untranslated region (5´ UTR), 3´ untranslated region (3´ UTR), downstream intergenic (within 300 bp of the 3´ UTR), and distal intergenic (>300 bp of the 3´ UTR) regions. (**B**) Distribution of MADS-box transcription factor binding sites relative to transcription start sites. (**C**) Functional enrichment of target genes of MADS-box transcription factors. The size of the point represents the count of target genes, and the gradual color change from red to blue represents the adjusted *P*-value change from low to high. (**D**) Downstream regulatory network of MADS-box transcription factors. Each point represents a gene, with colors indicating the group to which the gene belongs: MADS-box transcription factors, other transcription factors, or additional genes. The lines illustrate the regulatory relationships between gene pairs. (**E**) Heatmap of six genes was found to be co-regulated by *mads9* and *madsA*, with the *z*-score normalized expression levels indicated by the color bar (red indicating a relatively higher expression level and blue indicating a lower expression level). (**F**) Expression levels of four co-regulated genes by *mads9* and *madsA* during dynamic dimorphic transition. Red represents the transformation from the mycelial phase to the yeast phase (M to Y), and blue represents the transformation from the yeast phase to the hyphal phase (Y to M). The dots represent the normalized mean expression of three biological replicates at the same time, and the shading represents the standard error range. The horizontal axis is time points. TPM, transcripts per million.

Since *mads9* and *madsA* exhibit opposing regulatory effects by regulating distinct pathways, we further investigated whether there is potential crosstalk by performing ChIP-seq on FLAG-tagged *mads9* and *madsA* mutants under both hyphal and yeast conditions. The majority of *madsA* binding peaks were in promoter regions (82% in hyphal and 74% in yeast), while *mads9* showed significant binding in both promoter (60% in hyphal and 56% in yeast phases) and exon regions (Fig. 6A). Relative to the transcription start site, the binding peaks of *mads9* and *madsA* were mainly distributed within 1 kb around the transcription start site (Fig. 6B). Motif analysis identified enriched binding motifs consistent with genome-wide transcription factor binding profiles in both growth conditions (Table 1).

**TABLE 1:** Enrichment motifs for binding sites of MADS-box transcription factors

Motif	Gene	Phase	*E*-value
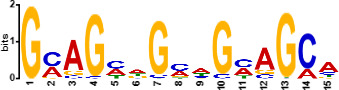	*mads9*	M	1.5e−028
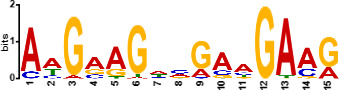	*mads9*	Y	5.5e−19
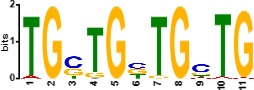	*mads9*	Y	1.2e−012
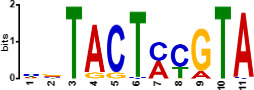	*madsA*	M	9.3e−108
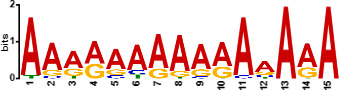	*madsA*	M	1.7e−048
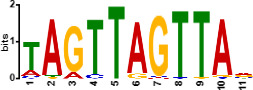	*madsA*	M	1.5e−020
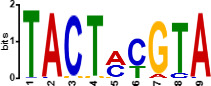	*madsA*	Y	2.7e−044
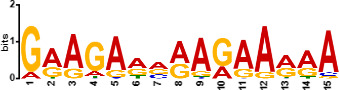	*madsA*	Y	2.0e−004

In the hyphal conditions, *mads9* target genes were enriched in “DNA binding transcription factor activity (GO:0003700)” and “zinc ion binding (GO:0008270),” while *madsA* targets were enriched in “DNA binding transcription factor activity (GO:0003700)” and “plasma membrane (GO:0005886)” (Fig. 6C). However, in the yeast conditions, significant enrichment was not observed for *mads9* targets, whereas *madsA* targets were enriched in “protein dimerization activity (GO:0046983)” (Fig. 6C), aligning with the above RNA-seq results.

Integrating the ChIP-seq and RNA-seq data revealed a set of downstream genes regulated by *madsA* and *mads9*. Specifically, under hyphal conditions, 296 genes were regulated by *madsA* and 26 by *mads9*, while in the yeast phase, 3 genes were regulated by *madsA* and 11 by *mads9* (Fig. 6D). Additionally, *Mp1p*-like protein 13 (*TM090004.00*) was identified as a downstream target of *mads9* in yeast. Given that *Mp1p* is a known virulence factor in *T. marneffei* (35), this suggests that *mads9* may play a role in the pathogenesis of the fungus.

We then constructed the regulatory networks associated with *madsA* and *mads9* in both hyphal and yeast forms to infer pathway crosstalk (Fig. 6D). We noticed six genes were found to be co-regulated by *mads9* and *madsA* under hyphal conditions (Fig. 6E), including pathogenesis-related protein 5 (*TM021467.00*), aspergillopepsin-2 precursor (*TM021553.00*), monosaccharide transporter (*TM050148.00*), general amino acid permease (*TM050341.00*), and two hypothetical proteins (*TM020572.00* and *TM011674.00*). Among them, four of the co-regulated genes showed the same trend in OE-*madsA* and KO-*mads9*, and they showed significant differential expression in the time-series transition data from our previous study (33) (Fig. 6F), suggesting that these genes may be downstream targets of *mads9* and *madsA* in regulating dimorphic transitions in different directions.

## DISCUSSION

In this work, we demonstrated that the MADS-box gene family plays a pivotal role in the morphological transitions of *T. marneffei*, an opportunistic human pathogen capable of thermal dimorphism. Through laboratory evolution combined with high-throughput sequencing, phylogenetic analysis and genetic manipulation, and ChIP-seq, we identify *mads9* and *madsA* as important regulators in the temperature-induced dimorphic transition and show that they are functionally oppositely divergent.

### MADS-box family regulation of dimorphic transition of *T. marneffei*

The ability of *T. marneffei* to switch from saprophytic hyphal forms to pathogenic yeast forms in response to temperature fluctuations is a sophisticated adaptation mechanism (6). This study identifies that the MADS-box transcription factors could fine-tune this morphological transition by expressing distinct genes. Specifically, the overexpression of *mads9* could delay the mycelium-to-yeast transition processes at elevated temperatures, while the overexpression of *madsA* could promote the yeast-to-mycelium transitions. In contrast, *mads10* did not appear to significantly contribute to dimorphic transition. This aligns with findings from other studies showing that MADS-box genes in fungi, such as *Candida albicans*, are implicated in morphogenesis and environmental response (27). Furthermore, the interaction of these MADS-box genes with downstream processes, including transmembrane transport and redox reactions, underscores their multifaceted roles in fungal physiology and adaptability.

### Functional divergence and gene evolution of MADS-box genes

The functional differences observed among the *mads9*, *mads10*, and *madsA* genes highlight the evolutionary dynamics within the MADS-box gene family. *Mads9* and *mads10* likely originated from a common ancestral gene, as evidenced by their structural similarities and the significant gene duplication observed within the family. However, their distinct regulatory functions in dimorphism reflect the process of neofunctionalization, where duplicated genes acquire new roles, thereby contributing to the organism’s adaptive potential.

In plants, similar patterns of gene duplication and functional divergence have been documented, particularly in the regulation of floral organ development (36–38). This suggests that the evolutionary pressures acting on MADS-box genes in fungi may parallel those in plants, driving the diversification of regulatory functions in response to environmental challenges. The complex interplay among these genes could further elucidate the evolutionary trajectory of MADS-box transcription factors and their roles in fungal pathogenicity.

### Temperature adaptation and environmental-to-pathogenic fungal transition

Thermodimorphism, where fungi switch between saprophytic hyphal growth at lower temperatures and yeast forms at human body temperature, is a critical adaptation that enables survival within the host (1). The ability of *T. marneffei* to undergo a temperature-induced dimorphic transition is a hallmark of its pathogenic potential, mirroring a broader evolutionary strategy employed by environmental fungi as they switch to opportunistic human pathogens. In this study, we revealed that the MADS-box transcription factors, particularly *mads9* and *madsA,* are involved in this transition with opposite functions. The enrichment of MADS-box genes in *T. marneffei* compared to other species within the *Talaromyces* genus suggests that gene duplication and functional diversification are important drivers of its ability to thrive in natural/host dual environments. This expands our understanding of how environmental fungi evolve to exploit host niches, a process that is becoming increasingly relevant as climate change alters ecosystems and increases human exposure to emerging fungal pathogens.

While our study provides evidence for the roles of *mads9* and *madsA* in regulating the dimorphic transition of *T. marneffei*, it is important to acknowledge the limitations of our current findings. Specifically, our experiments focused on 2 of the 15 MADS-box genes identified in the laboratory evolution, and the functional roles of the remaining genes in this family remain unexplored. Given that *T. marneffei* has a significant expansion of the MADS-box family compared with its closest relatives, these genes, although not enriched in laboratory evolution during the dimorphic transition, may also be involved in other morphological development and adaptation processes in *T. marneffei*. Additionally, comparative studies across different fungal species could provide insights into the evolutionary conservation and divergence of MADS-box gene functions in dimorphic transitions. Such efforts would not only deepen our understanding of the molecular mechanisms underlying fungal dimorphism but also pave the way for identifying potential therapeutic targets for fungal infections caused by *T. marneffei* and related pathogens.

## MATERIALS AND METHODS

### Strains and culture conditions

The wild-type *Talaromyces marneffei* strain PM1, isolated from a patient with culture-documented talaromycosis in Hong Kong (39), was utilized in this study. All strains (both mutants and wild-type) were cultured on Sabouraud dextrose agar (SDA; BD Difco) plates at 25°C or 37°C for indicated days, either to facilitate conidia collection or for phenotypic observation. For total RNA extraction, all strains were grown in Sabouraud dextrose broth (SDB; BD Difco) at 25°C or 37°C for 3 days before the next-step operation. For mycelium-to-yeast transition experiment, following 3 days of culture in SDB or 4 days on SDA plates at 25°C, samples were transferred to 37°C and continued growing. Time-course observations were conducted at indicated time intervals after temperature transition. Microscopic analysis was performed on the Primo Star microscope (Zeiss) equipped with the 20× objective (Plan-ACHROMAT, NA0.4, Ph2) or the upright microscope (Olympus, BX53) equipped with the 20× objective (UPLFLN20X, NA0.5). Images were captured with ZEN software and the cellSens Standard software bundled with Extend Focus Image acquisition module (Olympus).

### Isolation of mycelium, yeast, and conidia

The scraped spores, yeast culture, or mycelial culture were used to prepare a fungal suspension using phosphate buffered saline(PBS; pH 7.4, Gibco, cat. # 10010023). For yeast cells, the fungal suspension was filtered through four layers of Miracloth (Calbiochem, cat. # 475855), and the filtrate was collected in a new 50 mL centrifuge tube. For spores, it was filtered through a 40 µm cell filter (Falcon, cat. # 352340). No additional treatment was performed for hyphae. Then, the suspension was centrifuged at 10,000 × *g* for 5 min at 4°C. The supernatant was carefully removed using a 10 mL disposable pipette, leaving approximately 1 mL of liquid at the bottom of the tube. The cells were resuspended by gently tapping the tube. A 15 mL centrifuge tube containing 13 mL of 1× PBS solution was prepared, and the cell suspension was slowly added along the wall of the tube to minimize disturbance. The mixture was centrifuged at 100 × *g* for 1 min at 4°C, and the upper 12 mL of liquid was discarded, leaving 1 mL of concentrated fungal cells at the bottom for subsequent experiments.

### Adaptive laboratory evolution

The *T. marneffei* PM1 strain was inoculated into SDB and cultured at 32°C with agitation (180 rpm) for 7 days to induce the temperature-dependent morphogenetic transition from hyphae to yeast, a hallmark of its thermodimorphic behavior (7). Yeast-form cells were collected and re-inoculated into fresh SDB medium under the same conditions for an additional 3 days, followed by further isolation. After each passage, 10 µL of the culture was plated onto SDA agar plates and incubated at the same temperature for 7 days to assess growth morphology. If the strain predominantly exhibited yeast-form growth, the incubation temperature was decreased by 1°C, repeating the aforementioned step. When the temperature reached 25°C, a dimorphic transition defective strain was maintained in the yeast form at ambient temperature.

### Phylogenetic analysis of the MADS-box transcription factor family

Based on IPR protein domain annotation results (40), genes containing at least one MADS-box transcription factor-related domain (IPR002100, IPR033896, IPR033897, IPR036879) were screened from seven species of *Talaromyces* species and named as 1–15 in descending order according to gene length (Table S2), among which *mads7* corresponds to *madsA* in our previous studies (12). The identified MADS-box genes were subjected to multiple sequence alignment using MAFFT software (41) (v.7.407). Subsequently, a phylogenetic tree based on maximum likelihood was constructed using RAxML software (42) (v.8.2.12), with 1,000 bootstrap replicates and a Γ distribution for site rate variation, automatically selecting the amino acid substitution model based on the alignment results. The phylogenetic tree was visualized using EvolView software (v.2) (43).

### Genome synteny analysis

The protein sequences of *T. marneffei* were aligned with those of itself and six other *Talaromyces* species using BLASTp software (44), applying an *E*-value threshold of <1 × 10^−5^. Synteny regions in the PM1 strain’s genome were detected using MCScanX-transposed software with default settings (32). A Python script was used to organize synteny regions and identify those containing MADS-box transcription factors. The results of the genome synteny analysis were visualized using Circos software (45) (v.0.69). To distinguish segmental duplications from transposed duplications, MCScanX-transposed, a tool derived from the MCScanX algorithm, was utilized to classify gene duplication modes based on intra- and inter-species comparisons. Genes arranged in collinear clusters within *T. marneffei* were identified using MCScanX. These collinear gene pairs were classified as segmental duplications. For remaining dispersed paralogs, MCScanX-transposed compared target genes with outgroup species (related *Talaromyces* spp.) to detect syntenic regions. If paralogs lacked intra-species collinearity but showed synteny with outgroups, they were assigned ancestral loci. Paralogs lacking synteny in both intra- and inter-species comparisons were classified as transposed duplications.

### Preparation of gene overexpression strains

Genomic DNA was extracted from *T. marneffei*, and high-fidelity PCR was employed to amplify fragments of the coding region of the target gene for overexpression. The fragment of the promoter of the *gpdA* gene and the hygromycin resistance gene were amplified from the pEGFP-1 plasmid using high-fidelity PCR. These fragments were then assembled into an overexpression vector using the ClonExpress II One Step Cloning Kit (Vazyme). Strains were transformed with overexpression plasmids following a previously described protocol with modifications (46). Briefly, strains were cultured on SDA medium at 25°C for 7 days. Spores were collected by scraping with a fine toothpick, washed twice, and incubated for germination at 37°C with shaking at 150 rpm for 40 h. Germinated spores were centrifuged at 4,200 × *g* for 5 min, and excess supernatant was removed. Germinated spores were resuspended in 40 mL of enzymatic hydrolysis solution (β-glucuronidase and driselase, Sigma) at 37°C with shaking at 80 rpm until approximately 50% protoplasts were produced. The protoplasts were filtered using Miracloth and washed twice. Clean protoplasts were stored at −80°C until transformation. For transformation, a total of 5 µg of plasmid DNA was mixed with 1 × 10^6^ protoplasts and incubated on ice for 30 min. Subsequently, 1 mL of PEG-Tris-CaCl_2_ buffer (PTC; 60% PEG6000, 100 mM Ca^2+^) was added, and the mixture was incubated at room temperature for 60 min. Transformants were cultured on selective medium at 37°C for mutant selection and validated through whole genome sequencing.

### Preparation of gene knockout strains

The *mads9* knockout strains were acquired mainly based on the published method (47). In brief, the whole knockout constructs, including the Cas9 constructs, the gRNA constructs, and the gene deletion constructs, were electroporated into *T. marneffei,* and the transformants were subsequently screened by Sanger sequencing from colonies grown at 37°C. The Cas9 cassette was amplified from the vector pCas9P2AEGFP using primers FragC9-F and FragC9-R. The gene-specific gRNA designed with the CRISPR gRNA (guide RNA) Design Tool for Eukaryotic Pathogens (http://grna.ctegd.uga.edu/) was ligated into the pTmCas9 vector generated in our previous study (46), and the gRNA cassette was amplified using primer pairs TmU6P-F and Scaffold-R. The gene deletion constructs included the use of homologous arms (around 1.3 kb) with split marker gene that confers resistance to drug G418. In the first round of PCR, the 5′ homologous arm, the selection marker, and the 3′ homologous arm were amplified. In the second round of PCR, the 5′ and the 3′ arms were fused with the selection marker. In the third round of PCR, the full-length deletion construct was amplified.

The *mads10* knockout constructs contained only the *mads10* deletion constructs including 5′ and 3′ homologous arms (around 1 kb) with split hygromycin marker gene. For single gene knockout transformations, the wild-type PM1 strain was used. For *mads9* and *mads10* double gene knockout, the *mads10* knockout constructs were transformed into the KO-*mads9* strains. Primers used were indicated in Table S3. The knockout constructs were electroporated into competent *T. marneffei* conidia cells using Gene Pulser Xcell Total System (BIO-RAD). The positive transformants appeared on SDA plates supplemented with 200 µg/mL G418 or hygromycin at 37°C after growth for 2–3 days. For competent *T. marneffei* conidia cell preparation, the conidia cells pre-germinated in SDB for about 12–13 h at 25°C were collected initially. Then the cells underwent sequential washes, once with chilled ddH_2_O and twice with 1 M sorbitol (Sigma-Aldrich), and were finally suspended in 1 M sorbitol at a concentration above 10^9^ cells/mL.

### Verification of gene expression

For relative gene expression analysis, the cDNA was synthesized from total RNA (1 µg) using HiScript III 1st Strand cDNA Synthesis Kit (Vazyme, China). Diluted cDNA templates were amplified in ChamQ Universal SYBR qPCR Master Mix (Vazyme, China) using specific primer pairs designed for specific genes as listed in Table S3. The actin gene was used as an internal control. Three technical replicates in each test were carried out. Due to the difficulties in designing a primer pair which specifically targeted *mads10*, genotyping PCR using primer pair 10up-F and 10down-R could only be used for the *mads10* knockout strain identification. The positive *mads10* knockout transformant with selective marker inserted should produce a 4.3 bp DNA fragment, whereas the wild-type produced a 2.9 kb DNA fragment.

### Statistics of in dimorphic transition

(i) Maximum cell length on SDA under 37°C: the length of the main growth axis of each cell with or without branch in each field of view was measured, with a measured value of 1.513 corresponding to an actual length of 50 µm. Twenty fields of view (average 220 data) were analyzed for each strain at each time point. (ii) Hyphal tip expansion rate at 3.5 hpt: the expansion index was calculated as the ratio of the hyphal diameter at 3 µm away from the growth tip to the hyphal diameter at 30 µm away from the growth tip. A total of 200 hyphal tips were analyzed for each strain. (iii) Hyphal branching proximity index at 12 hpt: the distance from all distinguishable hyphal ends to the first branches or protrusions, as well as the distance between subsequent branches or protrusions, was measured. A total of 150 measurements were acquired for each strain. (iv) Hyphal branching distance index at 48 hpt: the distance between all distinguishable adjacent branches or protrusions along a hypha growth axis, as well as the distance from a hyphal tip to the neighboring branch or protrusion, was measured. Hyphae that were significantly reddened or had indistinguishable cell outlines were excluded from the analysis. A total of 450 measurements was acquired for each strain. All the above measurements were carried out using ImageJ bundled with 64-bit Java 8 (version 1.54 p) with Segmented or Freehand Line tool.

### RNA-seq library and data analysis

Total RNA of strains was extracted using the E.Z.N.A. fungal RNA kit (Omega Bio-Tek), following the manufacturer’s instructions with DNase I digestion to eliminate genomic DNA. The RNA concentration was measured using Nanodrop (Thermo Fisher Scientific Inc., USA), and the quality was assessed using Qsep_1_ (BiOptic, Inc., New Taipei City, Taiwan). RNA with a RIN > 8 was selected and stored at −80°C until downstream preparation. RNA-seq library construction and sequencing were performed on the Illumina XTEN platform by the Novogene Bioinformatics Institute (Beijing, China), generating paired-end 150 bp reads. Adapters and low-quality segments were trimmed from the raw sequencing data. The cleaned data were aligned to the PM1 reference genome (33) using HISAT2 (48), and gene expression transcripts per million matrices were generated with StringTie2 (49), correcting for sequencing depth and gene length. DEGs were identified by the DESeq2 package (50) with false discovery rate (FDR) < 0.05 and |log_2_(FC)| >1. Functional enrichment of genes was performed using the ClusterProfiler package (51).

### ChIP-seq library and data analysis

Strains were collected and frozen by immersion in liquid nitrogen. Frozen samples were ground and cross-linked with 1% formaldehyde for 10 min at room temperature with gentle rotation. Cross-linking was stopped by adding 200 mM final concentration of glycine solution in PBS, and cells were rotated at room temperature for 5 min. Fixed cells were pelleted at 1,500 × *g* for 10 min at 4°C and washed twice with 10 mL of ice-cold PBS and pelleted at 1,500 × *g* for 10 min at 4°C each time. ChIP-seq was performed using Anti-FLAG (DYKDDDDK tag rabbit mAb, CST#14793) at the Boyun Huakang Gene Technology Ltd. (Beijing, China). The resulting DNA fragments were purified with 1× DNA clean beads and subjected to library construction with the VAHTS Universal DNA Library Prep Kit for Illumina (v.3, ND607). The libraries were analyzed on the Agilent 2100 Bioanalyzer to assess fragment size distribution and sequenced on the Illumina Nova Seq 6000 (PE150) with reads varying across samples, ranging from 20,533,128 to 30,438,472.

The clean reads generated by ChIP-seq were evaluated using FastQC (https://github.com/s-andrews/FastQC) and aligned to the reference PM1 genome with Bowtie2 (52). Samtools (53) was then used to sort and index BAM files, and uniquely mapped reads were extracted using Sambamba (54). MACS2 (55) was employed to call peaks for each sample, using input DNA as a control. ChIP-seq peaks were annotated, compared, and visualized using the ChIPseeker (56) package, and motif analysis was conducted using MEME (57).

### Data analysis and visualization

All data analyses were performed using R (v.4.1.2). Visualizations were generated using ggplot2 (v.3.3.6), unless specified otherwise.

### Statistical tests

All statistical tests were conducted in R (v.4.1.2), with the relevant test methods and statistics indicated in the corresponding sections of the text.

## Data Availability

The pooled sequencing data for the dimorphic transition mutant strains of *T. marneffei* have been uploaded to NCBI under the project number PRJNA970557 (https://www.ncbi.nlm.nih.gov/bioproject/PRJNA970557/). The RNA-seq and ChIP-seq were uploaded to NCBI under the accession numbers GSE279912 (https://www.ncbi.nlm.nih.gov/geo/query/acc.cgi?acc=GSE279912) and GSE279913 (https://www.ncbi.nlm.nih.gov/geo/query/acc.cgi?acc=GSE279913).

## SUPPLEMENTAL MATERIAL

Fig. S1 msystems.00464-25-s0001.tif Results of mixed group analysis of *T. marneffei* dimorphism-defective population.

Fig. S2 msystems.00464-25-s0002.tif Genomic syntenic regions containing MADS-box genes in *T. marneffei*.

Fig. S3 msystems.00464-25-s0003.tif Gene expression level analysis of *T. marneffei* strains.

Fig. S4 msystems.00464-25-s0004.tif The phenotypes of *T. marneffei* strains grown on SDA plates at constant temperature.

Fig. S5 msystems.00464-25-s0005.tif The morphological changes of *T. marneffei* strains during the M-to-Y transition.

Supplemental legends msystems.00464-25-s0006.docx Legends for supplemental materials.

Table S1 msystems.00464-25-s0007.xlsx Annotation of mutation sites in genes and their upstream and downstream regions for dimorphism-defective strains in adaptive laboratory evolution.

Table S2 msystems.00464-25-s0008.xlsx Information on MADS-box family members in *Talaromyces* genus.

Table S3 msystems.00464-25-s0009.xlsx Primers used in this study.

ASM does not own the copyrights to Supplemental Material that may be linked to, or accessed through, an article. The authors have granted ASM a non-exclusive, world-wide license to publish the Supplemental Material files. Please contact the corresponding author directly for reuse.
