# Supplementary figures and images for: Functional diversification of the MADS-box gene family in fine-tuning the dimorphic transition of Talaromyces marneffei
Source: mSystems. 2025 Jun 3;10(7):e00464-25. doi: 10.1128/msystems.00464-25 (PMC12282165; doi:10.1128/msystems.00464-25)

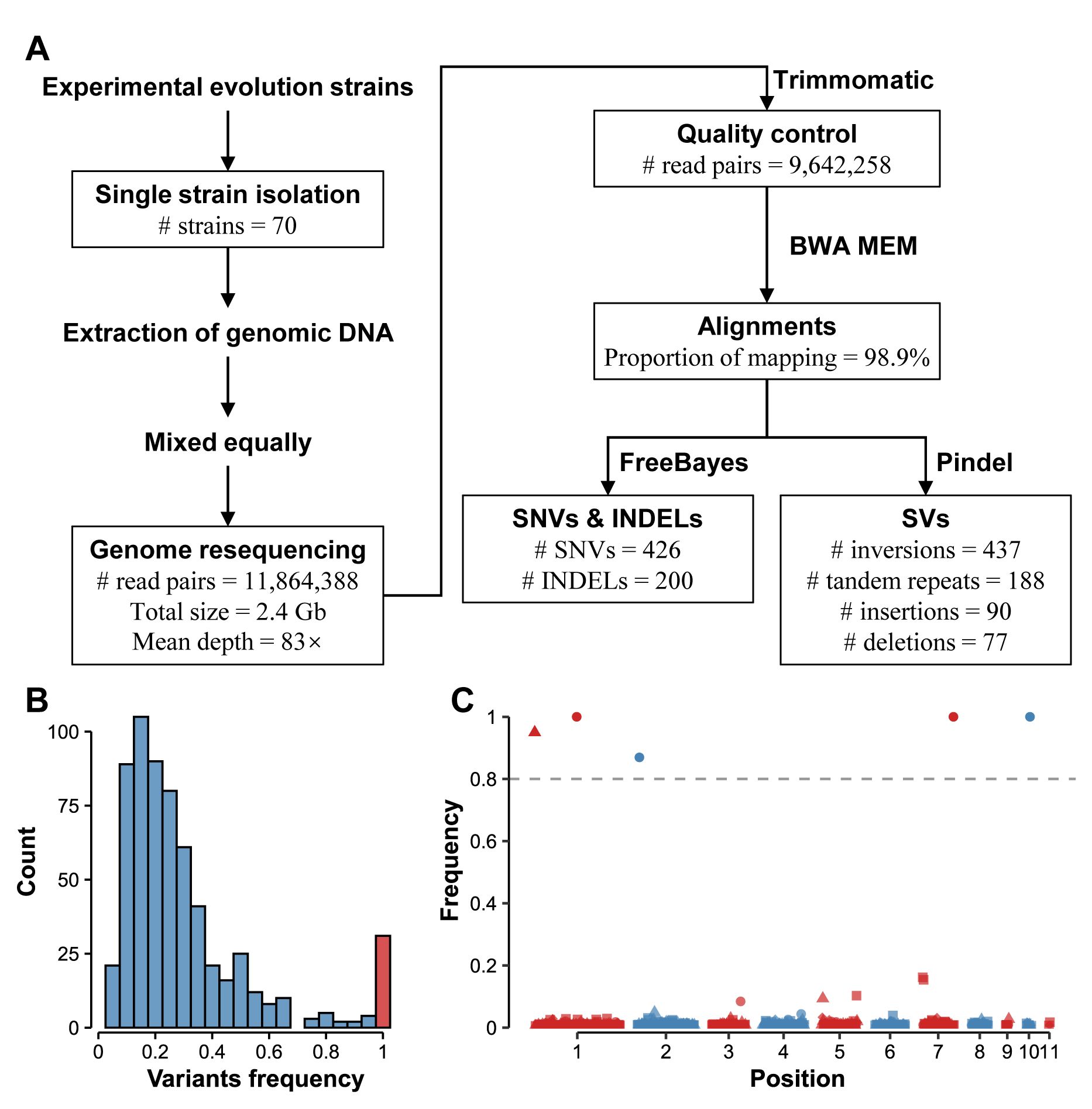

Supplement: Fig. S1 — Results of mixed group analysis of T. marneffei dimorphism-defective population. [file msystems.00464-25-s0001.tif]

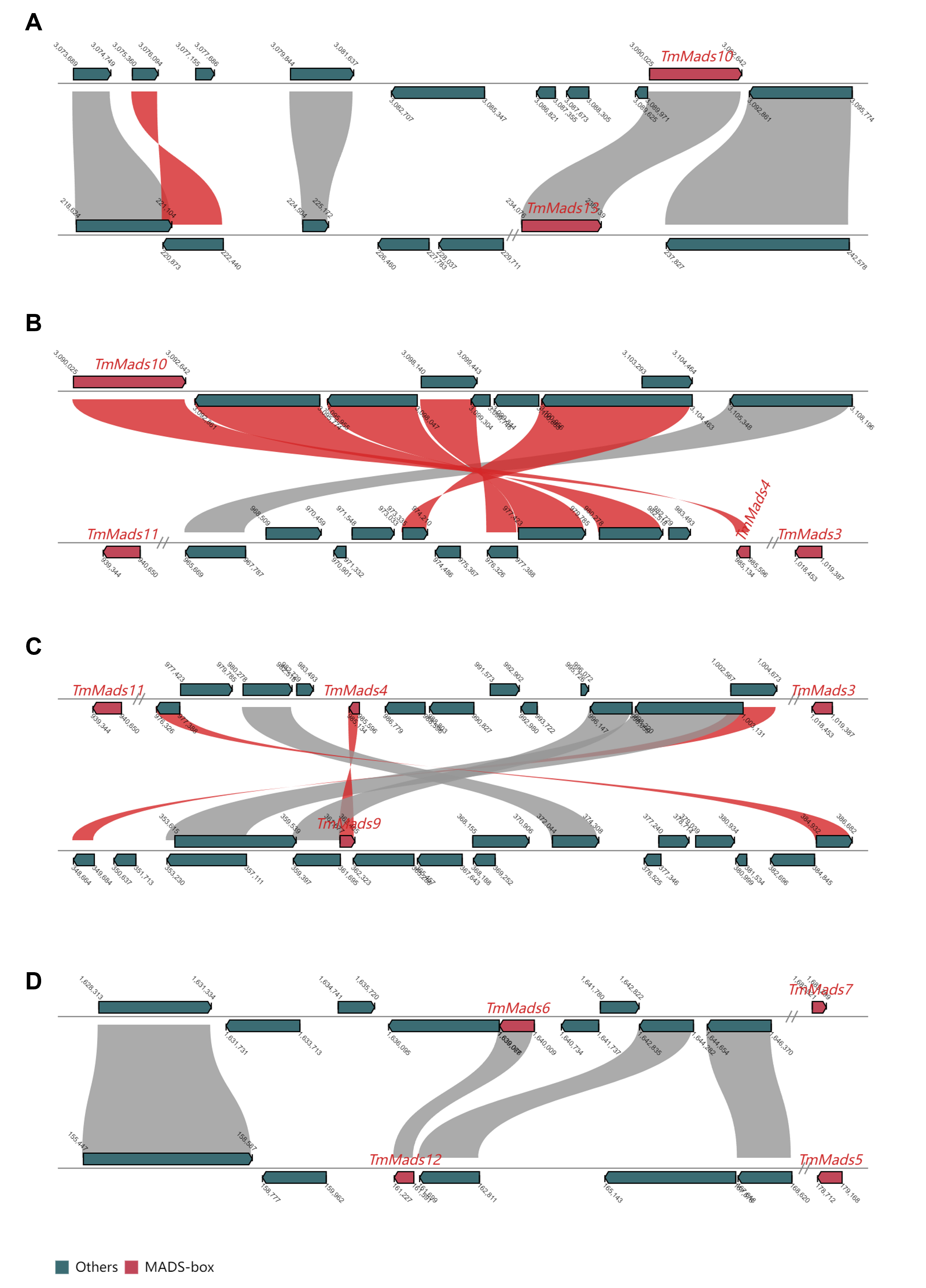

Supplement: Fig. S2 — Genomic syntenic regions containing MADS-box genes in T. marneffei. [file msystems.00464-25-s0002.tif]

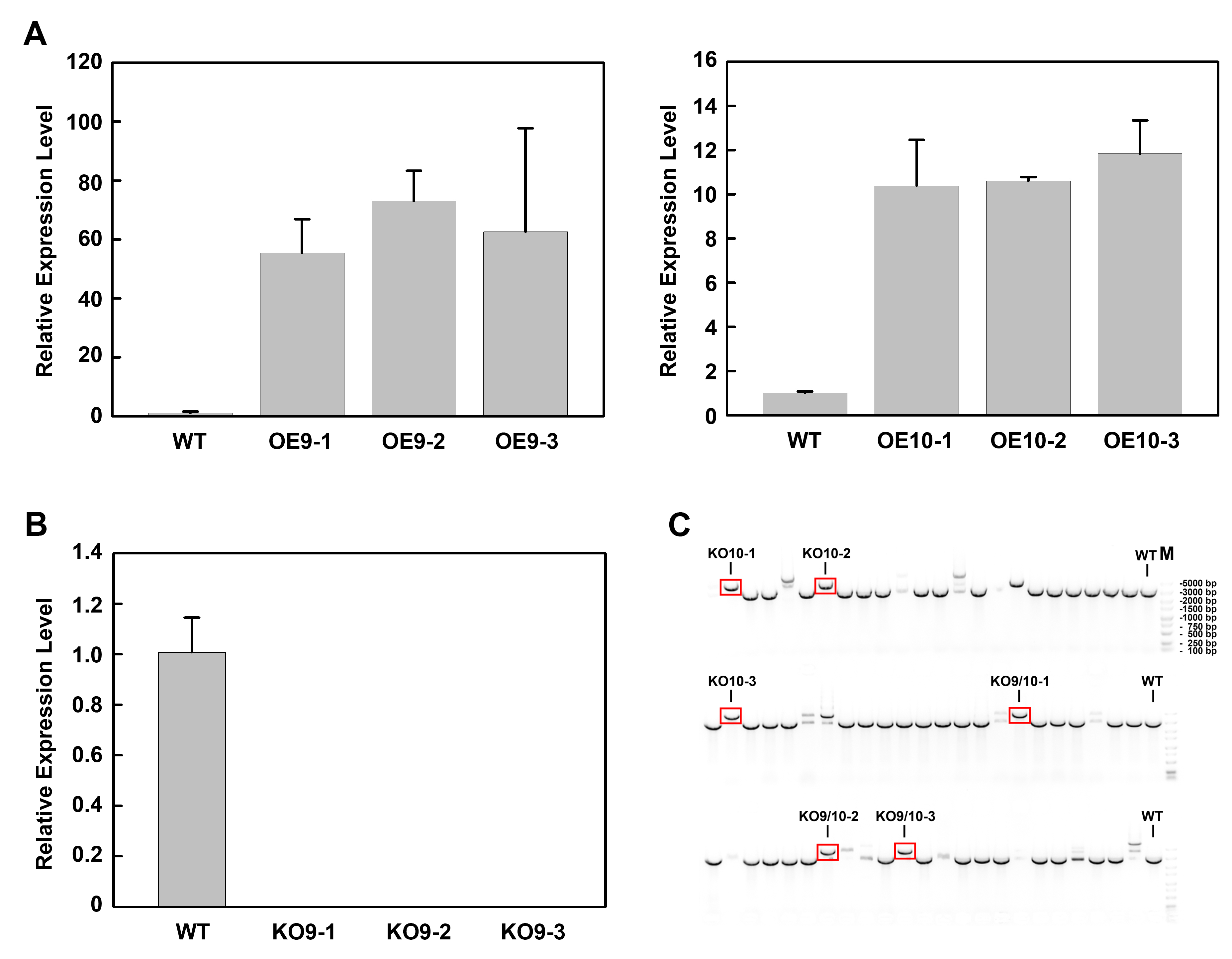

Supplement: Fig. S3 — Gene expression level analysis of T. marneffei strains. [file msystems.00464-25-s0003.tif]

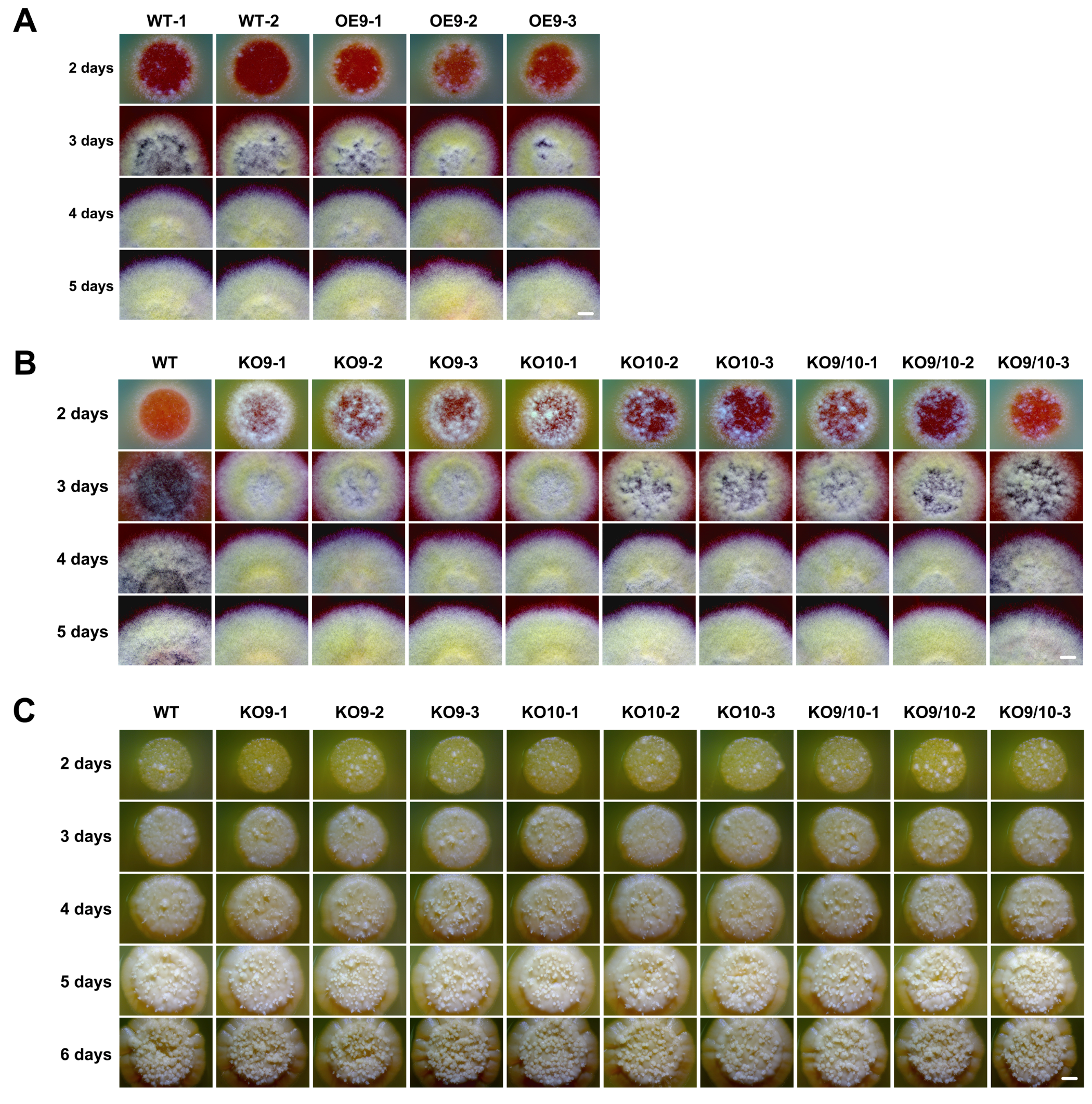

Supplement: Fig. S4 — The phenotypes of T. marneffei strains grown on SDA plates at constant temperature. [file msystems.00464-25-s0004.tif]

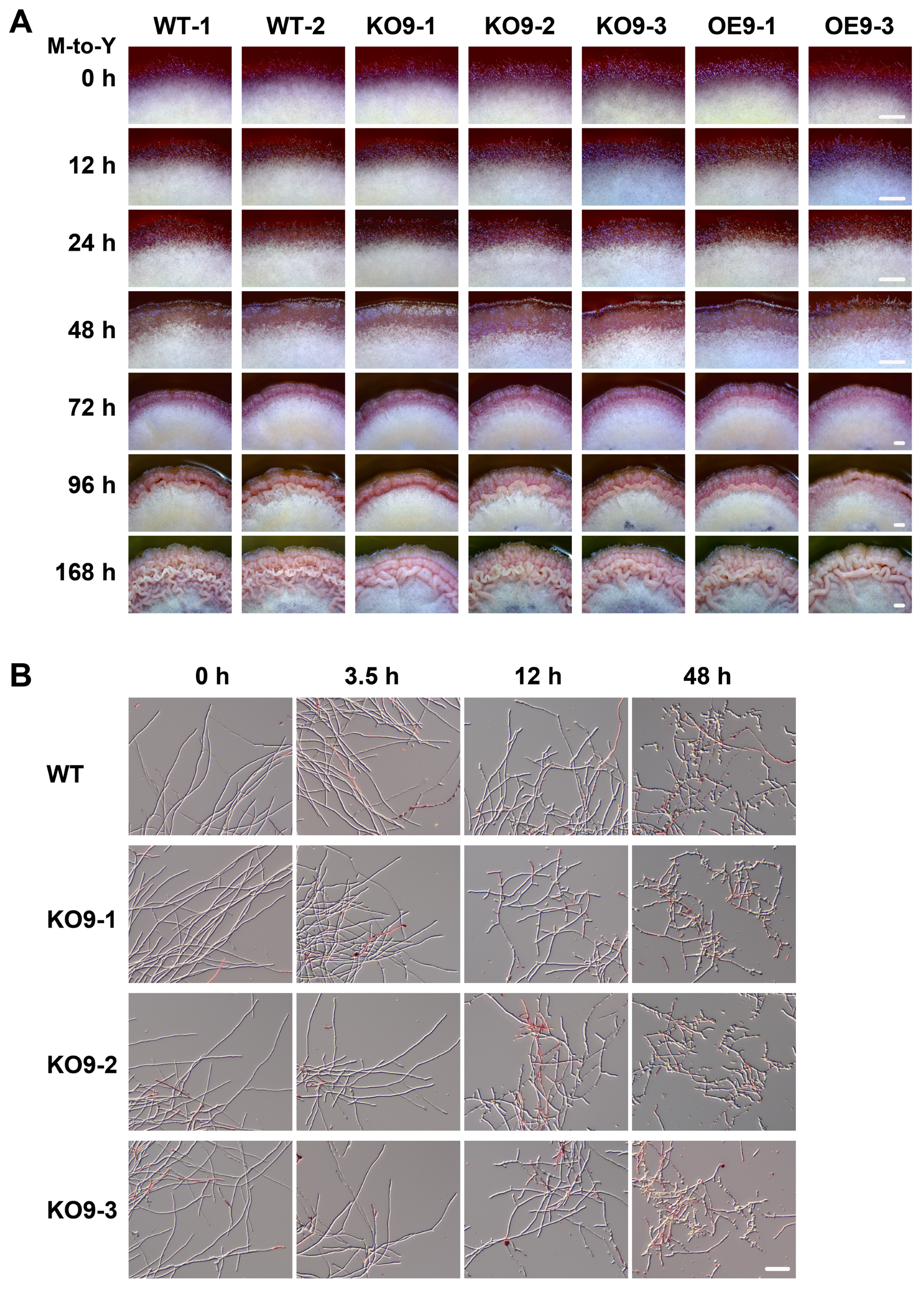

Supplement: Fig. S5 — The morphological changes of T. marneffei strains during the M-to-Y transition. [file msystems.00464-25-s0005.tif]
