# Supplementary material for: Functional diversification of the MADS-box gene family in fine-tuning the dimorphic transition of Talaromyces marneffei
Source: mSystems. 2025 Jun 3;10(7):e00464-25. doi: 10.1128/msystems.00464-25 (PMC12282165; doi:10.1128/msystems.00464-25)
Supplement: Supplemental legends — Legends for supplemental materials. [file msystems.00464-25-s0006.docx]

**Supplemental Figure 1 | Results of mixed group analysis of *T. marneffei* dimorphism-defective population.**

A) Flowchart of mixed group analysis of experimental evolution mutant population.

B) Frequency distribution of SNVs and INDELs in the genome of experimental evolution mutant population. The horizontal axis represents the frequency of SNVs INDELs in the mutants, and the vertical axis represents the number of mutation sites contained in the corresponding frequency. The red bar represents the number of sites with a mutation frequency of 100%.

C) Distribution of structural variation in the genome of experimental evolution mutants. The horizontal axis represents the position of the genome of *T. marneffei* PM1 strain, and 11 contigs in the assembly are distinguished by red and blue. The vertical axis represents the frequency of structural variation in the mutants, and the gray dotted line represents the structural variation screening threshold (structural variation mutation frequency exceeds 80%). The shape of the scattered points represents the type of structural variation: rectangles represent inversion mutations, triangles represent tandem duplications, diamonds represent fragment insertions, and circles represent fragment deletions.

**Supplemental Figure 2** | **Genomic syntenic regions containing MADS-box genes in *T. marneffei*.** Panels A-D illustrate four distinct genomic syntenic regions harboring MADS-box genes in *T. marneffei*. MADS-box genes are highlighted in red, with arrow directions indicating their transcriptional strand orientation. Connecting lines between genes represent anchor genes within syntenic regions: gray lines denote genes with consistent strand orientation, while red lines indicate genes with opposite orientations. The start and end positions of genes are labeled on the genome coordinates. Additionally, adjacent MADS-box genes located outside the syntenic regions are also depicted in Panels B-D.

**Supplementary Figure 3 | Gene Expression Level Analysis of *T. marneffei* Strains.**

A) Relative expression level of *mads9* and *mads10* in the overexpression strains compared with the wild-type.

B) Relative expression level of *mads9* in the knockout strains compared with the wild-type. Each of the test in qPCR included three technical replicates. Error bar represent mean±SE.

C) Genotyping results revealed the positive transformants of *mads10* knockout by homologous recombination. The highlighted DNA band indicted the bigger DNA fragment amplified (around 4.3 kb), representing positive transformants with successful insertion, while the amplified DNA fragments of the wild-type background were smaller (about 2.9 kb), indicating unsuccessful insertion.

**Supplemental Figure 4 | The Phenotypes of *T. marneffei* Strains Grown on SDA Plates at Constant Temperature.**

A) Comparison of the OE-*mads9* and the wild-type colonies grown at 25°C. Bar = 2 mm.

B) Comparison of the colonies of *mads9*, *mads10* and *mads9/mads10* knockout strains and the wild-type grown at 25°C. Bar = 2 mm.

C) Comparison of the colonies of *mads9*, *mads10* and *mads9/mads10* knockout strains and the wild-type grown at 37°C. Bar = 2 mm.

**Supplemental Figure 5 | The Morphological Changes of *T. marneffei* Strains During the M-to-Y Transition.**

A) Comparison of the colonies of *mads9* knockout and overexpression strains and the wild-type on SDA plates after transferred from 25°C to 37°C. Bar =1 mm.

B) Microscopic analysis of the morphological changes in the wild-type and the KO-*mads9* cells grown in SDB transferred from 25°C to 37°C at indicated time points. Bar = 50 μm.

**Supplemental Table 1 |** Annotation of mutation sites in genes and their upstream and downstream regions for dimorphism-defective strains in adaptive laboratory evolution.

**Supplemental Table 2 |** Information on MADS-box family members in *Talaromyces* genus

**Supplemental Table 3 |** Primers used in this study.
